# Disease evolution in systemic juvenile idiopathic arthritis: an international, observational cohort study through JIRcohort

**DOI:** 10.1186/s12969-023-00886-9

**Published:** 2023-09-07

**Authors:** M. Wallimann, K. Bouayed, E. Cannizzaro, D. Kaiser, A. Belot, E. Merlin, S. Poignant, C. Wouters, F. Hofer, T. Saurenmann, A. Koryllou, R. Carlomagno, M. Mejbri, M. Hofer, K. Theodoropoulou

**Affiliations:** 1 Department of Woman, Mother, Child, Unit of Pediatric Immunology, Allergology and Rheumatology, Lausanne University Hospital and University of Lausanne, Lausanne, Switzerland; 2 Department of Pediatrics, Unit of Rheumatology and Nephrology, Mother and Child University Hospital A. Harouchi, CHU Ibn Rochd, Casablanca, Morocco; 3 Faculty of Medicine and Pharmacy, Hassan II University, Casablanca, Morocco; 4 Department of Pediatrics, Unit of Pediatric Rheumatology, University Children’s Hospital, Zurich, Switzerland; 5 Department of Pediatrics, Unit of Pediatric Rheumatology, Childrens Hospital Lucerne, Lucerne, Switzerland; 6 Department of Pediatrics, Unit of Pediatric Nephrology, Rheumatology, Dermatology, Hospital Femme Mère Enfant, Hospices Civils de Lyon, Bron, France; 7 Department of Pediatrics, Clermont-Ferrand University Hospital, Clermont-Ferrand, F-63000 France; 8 Department of Pediatrics, University Hospital of Nantes, Nantes, France; 9 Department of Pediatrics, Unit of Pediatric Rheumatology, University Hospital Leuven, Louvain, Belgium; 10 Fondation Rhumatismes-Enfants-Suisse, Etoy, 1163 Switzerland; 11 Department of Pediatrics, Cantonal Hospital Winterthur, Winterthur, Switzerland

**Keywords:** Systemic onset juvenile idiopathic arthritis, Treatment, Biologics, Outcome, Predictors

## Abstract

**Background:**

Systemic juvenile idiopathic arthritis (systemic JIA) is a severe disease with both systemic and joint inflammation. This study aims to identify predictors of disease evolution within the systemic JIA population enrolled in the Juvenile Inflammatory Rheumatism cohort (JIRcohort).

**Methods:**

Observational patient cohort study with 201 recruited children from 4 countries (3 European, 1 North Africa) from 2005 until 2019, using retrospectively (2005–2015) and prospectively (2015–2019) routine care collected data.

**Results:**

Sixty-five patients with complete follow-up data for 24 months after first diagnosis were classified as monophasic (*n* = 23), polyphasic (*n* = 6) or persistent group (*n* = 36) corresponding to their evolution (unique flare, recurrent flares, or persistent disease activity respectively). The patients of the persistent group were more likely to have an earlier disease onset, before the age of 6 (OR 2.57, 95%-CI 0.70–9.46), persistence of arthritis at 12-months post-diagnosis (OR 4.45, 95%-CI 0.58–34.20) and higher use of synthetic DMARD (sDMARD, OR 5.28, 95%-CI 1.39–20.01). Other variables like global assessment by physician and by patient and C Reactive Protein levels at 12-months post-diagnosis were assessed but without any predictive value after adjusting for confounding factors.

**Conclusions:**

Our results suggest that the earlier disease onset, the persistence of arthritis throughout the first year of disease evolution and the need of sDMARD might predict a persistent disease course.

**Supplementary Information:**

The online version contains supplementary material available at 10.1186/s12969-023-00886-9.

## Background

Systemic juvenile idiopathic arthritis (systemic JIA) is a pediatric inflammatory condition classified within the spectrum of juvenile idiopathic arthritis (JIA). Systemic JIA is distinguished from other JIA subtypes due to its particularity of having a variety of systemic symptoms: persistent (> two weeks) undulating fever with a paroxysmal pattern, rash, lymphadenopathy, serositis (i.e. pericarditis, pleuritis or peritonitis) or organomegaly (hepato- and/or splenomegaly) [1]. Patients are also at risk of developing potentially fatal systemic inflammatory complications, such as macrophage activation syndrome, which has resulted in systemic JIA having the highest mortality rate (3.9/1000 person years) of all JIA subtypes [2, 3].

Despite recent medical advances, systemic JIA diagnosis is often challenging as it remains an exclusion diagnosis. Its heterogeneous and nonspecific clinical presentation, as well as the lack of specific laboratory markers, create difficulties for a rapid and accurate diagnosis. Systemic JIA reportedly accounts for 10% of all JIA cases in Europe, however its impact is likely under-represented due to the current classification criteria which have been criticized [4, 5]. New preliminary classification criteria have been recently proposed by an international consensus [6].

Clinical presentation of systemic JIA was studied by Behrens et al. in 136 children. The most frequent symptoms at first presentation were fever followed by arthritis and cutaneous rash, all diagnosed in over 75% of the children included. Lymphadenopathy, organomegaly and pericarditis were seen in much fewer patients. Articular involvement was oligo- or polyarticular in over 80% of cases, whereas the most frequent joints involved were the wrists, knees and ankles [7].

Disease evolution may be variable in systemic JIA. Three disease evolutive patterns were first described by Lomater et al. as either monocyclic, intermittent or persistent [8]. Singh-Grewal et al. are one of the few groups to examine the predictors of the disease evolution in systemic JIA. According to them, the only factor at diagnosis that is predictive of a non-monophasic (i.e. intermittent or persistent) evolution was the presence of a polyarticular arthritis. They described additional predictors of a non-monophasic course at three months (i.e. fever and active arthritis) and six months post diagnosis (i.e. erythrocyte sedimentation rate, ESR > 26 mm/hour and the persistent use of corticosteroids) [9]. Spiegel et al. identified thrombocytosis and persistent active systemic disease (six months after onset) as strong predictors for a poor functional outcome [10]. Persistent active systemic disease has proven to be a particularly important prognostic marker [8, 11]. So far, our knowledge of the early predictors in systemic JIA disease evolution is based on very limited data, which is fractured across groups with varying diagnostic definitions.

This international cohort study aimed to identify predictors of systemic JIA disease evolution in a real-life condition. We provided detailed diagnostic phenotypes of 65 enrolled patients including clinical, laboratory and treatment variables. We then examined univariate and multivariable correlations of these variables with the disease progression.

## Methods

### Cohort and study design

**Fig. 1:**
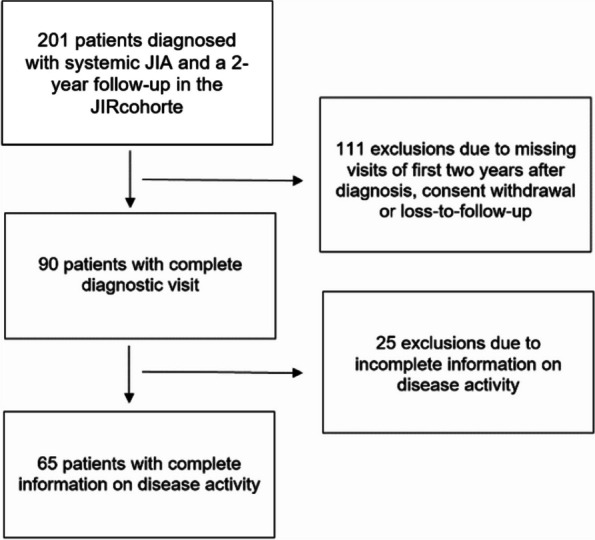
Flow diagram for inclusions and exclusions. JIA: juvenile idiopathic arthritis

An international multicenter observational patient cohort study with retrospectively and prospectively routine care collected data was conducted on systemic JIA patients enrolled in the Juvenile Inflammatory Rheumatism cohort (JIRcohort) with a minimum follow-up of two years. Patients were considered eligible if diagnosed with systemic JIA according to the ILAR criteria [1] and/or an expert opinion and if complete information on disease activity was available. The study size is described in Fig. 1. Signed informed consent was provided by all legal guardians and by older children with age-adapted consent forms. The JIRcohort is an observational inception cohort study developed to promote multicentric international studies on juvenile inflammatory rheumatisms aiming a better understanding of these rare diseases and their therapies. The JIRcohort has been approved by the ethics committee of the canton of Vaud (CER-VD) with the following number: 2018–02161 (date: 19.08.2013).

### Data collection

This study contained routine care data from 2005 until October 2019, collected retro- (2005–2015) and prospectively (2015–2019). Eleven centers in Europe and North Africa contributed to the data collection in this study. The 65 included patients were recruited and followed at the participating pediatric rheumatology tertiary referral centers (Switzerland: Romandie (*n* = 17), Lugano (*n* = 1), Winterthur (*n* = 1), Zurich (*n* = 5); Belgium: Leuven (*n* = 9); France: Besancon (*n* = 1), Clermont-Ferrand (*n* = 5), Versailles (*n* = 1), Nantes (*n* = 1), Lyon (*n* = 11); Morocco: Casablanca (*n* = 13)).

### Assessment of variables for outcome definition

Disease evolution was assessed at 6 and at 12 months post-diagnosis and then annually, following the assessment of the disease status by the pediatric rheumatologist (i.e. classification into either inactive disease, continued activity or flare). Clinically inactive disease was defined using the absence of systemic symptoms (fever, rash, organomegaly, or generalized lymphadenopathy), absence of active arthritis and uveitis, normal erythrocyte sedimentation rate (ESR) or C-reactive protein (CRP) results, physician’s global assessment (PGA) of disease activity of 0 on a score ranging from 0 (no disease activity) to 10 (worst disease activity) and duration of morning stiffness of ≤ 15 min [12]. The inflammatory markers were defined as elevated if CRP > 10 mg/L and ESR ≥ 26 mm/h according to previous studies [9]. A flare was defined as reoccurrence of one of the previously mentioned variables (e.g. systemic symptoms, active arthritis, elevation of inflammatory markers, a qualified physicians assessment). Remission was defined as twelve months of clinically inactive disease without any treatment, or 6 months of clinically inactive disease under treatment [13]. The monophasic disease course in this study was defined as the occurrence of disease (systemic symptoms and/or arthritis) followed by a remission without relapse within 2 years of follow up. The polyphasic course was defined by the recurrence of disease at any time after having achieved inactive disease off medication. A persistent evolution was defined as the presence of systemic symptoms and/or arthritis and/or abnormal laboratory results at all three follow-up time points, i.e. 6 months, 12 months and 24 months post-diagnosis. The main outcome variable for the present analysis was type of disease evolution at 24 months with the following two categories: persistent versus non-persistent disease evolution, the latter including both the monophasic and polyphasic disease course.

### Assessment of potential predictors

Clinical, laboratory and demographic variables were defined according to results from previous studies and the judgement by the clinical experts and were evaluated as potential predictors of disease evolution [7, 9, 11, 14]. Clinical variables included arthritis (oligoarthritis, polyarthritis), fever, rash, lymphadenopathy, hepatomegaly, splenomegaly, serositis, physician’s global assessment (PGA) for disease activity, assessment of disease activity by patient using the visual analogue scale (VAS), use of biologic disease modifying anti-rheumatic drugs (bDMARD), synthetic disease modifying anti-rheumatic drugs (sDMARD) and glycocortidoids. Laboratory variables comprised C-recative protein (CRP) and erythrocyte sedimentation rate (ESR). Demographic variables incorporated sex, age at diagnosis, age at disease onset, time from onset of symptoms to diagnosis and time to inactive disease. Arthritis and systemic symptoms were described by the physician at the appointment. The inflammatory markers were defined as elevated if CRP > 10 mg/L and ESR ≥ 26 mm/h accordingly to previous studies [9]. The disease activity was assessed by physician using the Physician Global Assessment of disease activity score (GPA) and by patient using the Visual Analogue Scale, both from 0 to 10 [12].

### Statistical methods

Absolute and relative frequencies were calculated for the description of results from categorical variables. Continuous variables were represented by their medians and interquartile range (i.e., 25^th^ and 75^th^ percentiles). Categorical variables were expressed as total numbers (n) and percentages (%). Analysis of variance (ANOVA) was performed to assess differences of clinical, laboratory and therapy features between the three disease evolution groups (i.e. monophasic, polyphasic and persistent). Univariate and multivariable logistic regression were performed to assess the association between each potential predictor and the outcome of persistent disease evolution at 24 months after first diagnosis. A backward stepwise approach regression was performed excluding the following variables: CRP, GPA physician at 12 months (due to the large amount of missing data) and treatment duration variables to avoid exclusion of patients without treatment. Associations were reported as crude and adjusted Odds Ratios (OR) with corresponding 95% confidence intervals and *p*-values (the level of statistical significance was set at 5%). For the statistical analysis we used IBM SPSS Statistics for Windows, version 29.0. Armonk, NY.

## Results

### Demographic characteristics: earlier disease onset in the persistent disease evolution group

Two thirds of the eligible patients had to be excluded from the present analyses due to missing visits, consent withdrawal, loss of follow-up, or incomplete information on disease activity (Fig. 1). Of those included, a third was classified as monophasic (35.4%), a small fraction as polyphasic (9.2%), and the majority as persistent (55.4%) corresponding to their disease evolution. The included subjects were more often girls and tended to have more often arthritis and fever compared to excluded patients. The median age at disease onset was significantly younger in the persistent group compared to the monophasic and polyphasic groups (*p* = 0.029, ANOVA). The median time to diagnosis in the persistent group (2 months) was about 1 month longer compared to the monophasic and polyphasic group; *p* = 0.252, ANOVA). Further demographic characteristics are shown in Table 1.

**Table 1:** Demographic and clinical characteristics of study cohort and excluded patients

	**Inclusions (all)**	**Monophasic**	**Polyphasic**	**Persistent**	**Exclusions**
**Number of patients**	65	23	6	36	136
**Female, number**	41 (63%)	18 (78%)	4 (80%)	19 (51%)	69 (48.6%)
**Age at diagnosis in years**	4.8 (2.4 – 8.5)	7.3 (1.9 – 10)	7 (2.9 – 13)	4.5 (2.6 – 6.5)	4.5 (2.2 – 9.3)
**Age at disease onset**	4.6 (2.2 – 8.2)	7.25 (1.6 – 10)	7 (2.6 – 13)	3.9 (2.3 – 6.5)	4.5 (2.2 – 9.3)
**Time from onset of symptoms to diagnosis in months**	1.1 (1 – 4.1)	1 (0.6 – 2)	1.1 (0.75 – 3.25)	2 (1 – 6.75)	1.8 (0.75 – 4.5)
**Time to inactive disease from diagnosis in months**	14 (3.5 – 37.5)	7 (2 – 14)	7 (0.75 – 17.25)	28 (6 – 72.25)	Not assessed
**Time to follow-up in years**	4.7 (3.1 – 8.3)	3.7 (2.7 – 5.8)	4.1 (3 – 5.3)	6.3 (3.6 – 9.6)	Not assessed
**Arthritis**	59 (91%)	18 (74%)	5 (83%)	35 (97%)	113 (83%)
**Oligoarthritis**	29 (45%)	11 (48%)	3 (50%)	14 (39%)	39 (29%)
**Polyarthritis**	29 (45%)	6 (26%)	2 (33%)	21 (58%)	72 (53%)
**Fever**	64 (99%)	23 (100%)	6 (100%)	35 (97%)	117 (86%)
**Rash**	46 (71%)	18 (78%)	4 (67%)	24 (67%)	106 (78%)
**Lymphadenopathy**	26 (41%)	9 (41%)	2 (33%)	15 (42%)	58 (43%)
**Hepatomegaly**	11 (17%)	4 (17%)	1 (17%)	6 (17%)	24 (18%)
**Splenomegaly**	14 (22%)	8 (35%)	1 (17%)	5 (14%)	22 (16%)
**Serositis**	12 (19%)	2 (9%)	2 (33%)	8 (23%)	21 (15%)

All age and time ranges are reported by median (25th-75th percentiles). Oligoarthritis is defined as arthritis affecting less than five joints. Polyarthritis is defined as arthritis affecting five or more joints

### Clinical characteristics: polyarthritis at diagnosis in over half of all children with persistent disease evolution and persistence of arthritis at 12 months of disease course

Initial clinical presentation was characterized by fever for all three disease evolution groups. Other systemic symptoms such as cutaneous rash and splenomegaly were less frequent but showed differences in prevalence between the three evolution groups; splenomegaly trend to be most prevalent in the monophasic disease evolution group and serositis in the non-monophasic groups. Arthritis at diagnosis was seen in nearly all patients from the persistent disease evolution group, half of them presenting with polyarthritis (Table 1). However, statistically significant differences were not observed in the clinical presentation at diagnosis among the three groups.

At time point “6 months”, significant differences between the three evolution groups (i.e. monophasic, polyphasic and persistent) were seen for having fever (0%, 33% 0% respectively; *p* < 0.001, ANOVA), in the median number of active joints (0, 1, 0 joint respectively; *p* < 0.001, ANOVA) and in the mean value of disease activity evaluated by physician (GPA 0, 4, 2; *p* = 0.013, ANOVA) as well as by patient (VAS 0, *missing, 7; *p* < 0.001, ANOVA).

At time point “12 months”, significant differences between the monophasic, polyphasic and persistent group were found in the mean number of active joints (0, 0, 2.5 joints; *p* = 0.007, ANOVA), the disease activity scored by physician (GPA 0, 0, 5; *p* < 0.001, ANOVA) as well as by patient (VAS 0, 0, 4.5; *p* = 0.001, ANOVA).

### Laboratory characteristics: higher C-reactive protein (CRP) and erythrocyte sedimentation rate (ESR) values in the polyphasic and persistent groups

Inflammatory laboratory markers, such as ESR and CRP, varied across disease groups (Table 2). Median ESR and CRP values were the highest in the polyphasic group at diagnosis and at 6 months after diagnosis. However, at 12 months, the mean CRP value was significantly higher in the persistent group compared to the monophasic and the polyphasic group.

**Table 2:** Laboratory characteristics of the study population (*N* = 44)

	**Erythrocyte Sedimentation Rate [mm/h]**	**C-Reactive Protein [mg/L]**
**At diagnosis**	*N* = 38	*N* = 44
** Monophasic (*n* = 15 / *n* = 17)**	74 (13 – 90)	85.5 (52.6 – 127.2)
** Polyphasic (*n* = 2 / *n* = 3)**	125 (120 – *)	123 (119 – *)
** Persistent (*n* = 21 / *n* = 24)**	86 (43.5 – 100)	107 (77.3 – 150)
* p*-value	0.564	0.084
**6 months after diagnosis**	*N* = 29	*N* = 37
** Monophasic (*n* = 13 / *n* = 17)**	8 (4 – 10.5)	2 (0.15 – 8.5)
** Polyphasic (*n* = 1 / *n* = 4)**	100 (100 – 100)	96.5 (0.4 – 310.5)
** Persistent (*n* = 15 / *n* = 16)**	11 (5 – 40)	3 (0.9 – 10.8)
* p*-value	< 0.001	< 0.001
**12 months after diagnosis**	*N* = 37	*N* = 44
** Monophasic (*n* = 12 / *n* = 14)**	5 (3.25 – 20.5)	0.8 (0.15 – 1.2)
** Polyphasic (*n* = 4 / *n* = 5)**	14 (4 – 64.5)	1.0 (0.3 – 92)
** Persistent (*n* = 21 / *n* = 25)**	9 (6 – 43.5)	23.2 (6.9 – 80.5)
* p*-value	0.307	0.041

All age and time ranges are reported by median (25th-75th percentiles). *75th percentile could not be calculated with only 2 ESR and 3 CRP values available respectively

*P*-values are calculated using ANOVA

### Therapeutic characteristics: higher treatment frequency with synthetic disease modifying anti-rheumatic drugs (sDMARD) in the persistent disease evolution group. Higher use of tocilizumab in the non-monophasic groups

Nearly all patients were treated with glucocorticoids (87% of the monophasic, 100% of the polyphasic and 95% of the persistent group; *p* = 0.449, ANOVA). All children in the polyphasic group, 94% in the persistent group and 70% in the monophasic group were treated with biologic agents (*p* = 0.014, ANOVA). The biologic agents (bDMARD) used in this study cohort were abatacept (*n* = 1; CTLA4-Ig), adalimumab (*n* = 1; Anti-TNF-α), anakinra (*n* = 32; Anti-IL 1 receptor-antagonist), canakinumab (*n* = 25; Anti-IL1β monoclonal antibody), etanercept (*n* = 10; Anti-TNF-α), Rituximab (*n* = 1; Anti-CD20 antibody), tocilizumab (*n* = 33; Anti-IL6R monoclonal antibody). Tocilizumab showed a significant difference in the treatment frequency in the three disease evolution groups (17,4%, 66,7% and 69,4% in the monophasic, polyphasic and persistent group, respectively; *p* < 0.001, ANOVA). In the persistent group, tocilizumab was used as first line therapy in 14 of the 25 treated patients (56%). In 11 patients (44%) it was used second line, either after anti-IL-1 treatment (9 patients, 81.8%) or after anti-TNF- α treatment (2 patients, 18.2%). Anti-IL-1 biologics (anakinra and canakinumab) were highly used in this study cohort (*n* = 57). But no statistically significant difference between the three disease evolution groups could be identified neither for the use of anakinra nor for the use canakinumab. Significant difference was seen in the frequency of sDMARD used (26%, 67% and 83% of the patients in the monophasic, the polyphasic and the persistent group, respectively; *p* < 0.001, ANOVA). Detailed information on treatment duration is shown in Table 3.

**Table 3:** Type and duration (median and interquartile range in years) of treatments among study patients (*N* = 60)

	**Monophasic**	**Polyphasic**	**Persistent**	*P*-value
**bDMARD (*N* = 56)**	2.2 (0 – 3.9)	4.25 (2.5 – 5.4)	5.1 (3.4 – 8.5)	0.014
**Anakinra (*N* = 32)**	1.15 (0.3 – 5.0)	2.95 (0.9 – 3*)	1.1 (0.6 – 2.4)	0.936
**Canakinumab (*N* = 25)**	2.1 (1.0 – 3.6)	3.6 (1.1 – 3.6*)	3.8 (0.9 – 6.0)	0.761
**Tocilizumab (*N* = 33)**	3.4 (2.9 – 4.6)	3.3 (1.0 – 4.5)	4.7 (2.5 – 6.6)	0.012
**Glucocorticoids (*N* = 60)**	0.5 (0.2 – 1)	1.1 (0.4 – 4.3)	2.6 (1 – 4.9)	0.037
**sDMARD (*N* = 40)**	0 (0 – 0.05)	0.3 (0 – 0.95)	4.1 (0.9 – 6.1)	< 0.001

Median (25–75 percentiles) if not otherwise specified. *50 percentiles

*bDMARD* Biologic disease modifying anti-rheumatic drugs, *sDMARD* Synthetic disease modifying anti-rheumatic drugs

*P*-values are calculated using ANOVA

### Predictors of a persistent disease evolution

The patients of the persistent disease evolution group trended to have the disease onset at a younger age. In univariate analysis, disease onset before the age of 6 years was associated with an increased likelihood estimation for a persistent disease evolution according to the crude OR. However, this association lost its significance after adjusting for the other potentially influencing factors (Table 4).

**Table 4:** Univariate and multivariable logistic regression analysis of factors predicting a persistent compared to a non-persistent disease evolution (monophasic and polyphasic were combined to one category)

**Potential predictors**	**Persistent disease evolution group**^**a**^ **(% of patients)**	**Crude odds ratio (95% CI)** ***N* = 65**	*P*-value	**Adjusted odds ratio** **(95% CI)** ***N* = 65**	*P*-value
**Age at disease onset < 6 years (vs. age ≥ 6 years,ref.categ.)**	72.2%	3.68 (1.30 – 10.40)	0.014	2.57 (0.70 – 9.46)	0.155
**Arthritis at 12 months (vs. no arthritis, ref. categ.)**	44.5%	2.11 (1.24 – 3.6)	0.006	4.45 (0.58 – 34.20)	0.151
**bDMARD use**	94.4%	5.41 (1.03 – 28.46)	0.046	1.35 (0.15 – 11.85)	0.785
**sDMARD use**	83%	15.8 (4.68 – 53.79)	< 0.001	5.28 (1.39 – 20.01)	0.014
**PGA ≥ 3 at 12 months**	73.7%	0.02 (0.002 – 0.21)	< 0.001	^b^	
**CRP > 10 mg/L at 12 months**	47.2%	19.1 (3.54 – 103.16)	< 0.001	^b^	

Median (25–75 percentiles) if not otherwise specified

*bDMARD* Biologic disease modifying anti-rheumatic drugs, *sDMARD* Synthetic disease modifying anti-rheumatic drugs, *PGA* Physician’s Global Assessment, *CRP* C-reactive protein

^a^ Persistent disease evolution group; persistent disease during 24 months

^b^ Not included in the final model

Active arthritis was significantly more often seen in the persistent group at 12 months of diagnosis (44.5% vs. 6.9%, *p* = 0.001, supp Table 1). However, after adjusting for potential confounding this presumed predictive factor was no longer statistically significant (Table 4). Detailed information on clinical and laboratory features comparing persistent to non-persistent disease evolution are shown in Supplementary Table 1.

The treatment frequency with bDMARD and sDMARD was higher in the persistent group (94.4% vs. 75.9%, *p* = 0.046; 83% vs. 34%, *p* < 0.001). In the bDMARD group, tocilizumab was more used in the persistent disease evolution group (69.4% vs. 27.6%, *p* = 0.001). Glucocorticoids were highly used by patients with both persistent and non-persistent disease evolution (95% vs. 90%, respectively, *p* = 0.478). After adjusting for potential confounding our multivariable analysis showed that ‘treatment with sDMARDs’ was the strongest predictor for a persistent disease evolution (Table 4).

## Discussion

Systemic juvenile idiopathic arthritis is a chronic disease that can remain active over several years resulting in significant morbidity for young adults with an impact on their social, professional and financial future. Although it has been established that there are three different types of disease evolution, information on the predictors of the disease course is limited. The present study was designed to describe the occurrence and clinical course of the different disease evolution types as well as to investigate the prognostic capacity of clinical features to better predict a chronic evolution.

First, these results show that our systemic JIA population presented all the three previously described disease evolution types: monophasic, polyphasic and persistent evolution. More than half of the patients were classified as having persistent disease (55.4%), followed by the monophasic group (35.4%), with only a small portion having a polyphasic evolution (9.2%). These findings are in line with the results from a prospective observational study that analyzed 45 patients treated in an academic rheumatology center in Canada between 1996 and 2000 [9], but differ from a more recent retrospective study including 76 patients treated in Boston Children’s Hospital between 1996 and 2015 were lower percentage of persistent disease course was reported [15]. Because of the low number of patients in the polyphasic group, this group was combined with the monophasic one (non-persistent group) for the assessment of potential predictors for a persistent disease course.

Demographic features, such as age at disease onset, sex and time to follow-up, are consistent with findings in previous studies [7, 11]. Concerning clinical presentation, most children in this cohort presented initially with fever and/or arthritis, while other systemic symptoms such as cutaneous rash, lymphadenopathy, organomegaly or serositis were less frequent. These results are in agreement with a retrospective observation study that included 136 patients diagnosed with systemic from three tertiary rheumatology referral centers in the United States of America between 1990 and 2005, as well as with the previously reported retrospective study including 76 patients treated in Boston Children’s Hospital besides rash that was among the most common symptoms following fever and arthralgia in this systemic JIA cohort [7, 15].

Importantly, early disease onset (< 6 years old) was significantly more frequently observed in the persistent group and was identified as a presumed predictor of persistent disease evolution, in line with the results of Flato et al. indicating early disease onset as a risk factor for poor outcome in JIA [16]. However, after adjusting for potential confounders this association was no longer statistically significant, highlighting the need of larger studies to address this question.

Furthermore, we observed a higher prevalence of polyarthritis in the persistent group during the first 6 months of disease evolution, with the patients among this group being more likely to have active at 12 months. In addition, the visual analogue scale (VAS) score at 12 months evaluated by patient and the physician’s global assessment (PGA) was significantly higher in the persistent group, reflecting the increased disease activity and severity in the persistent disease evolution group. Regarding the systemic features, our data show a higher trend of rash and splenomegaly in the non-persistent group at diagnosis, with, on the other hand, a higher frequency of fever, lymphadenopathy and rash in the persistent group at 12 months, but without any statistical significance.

The duration of elevated inflammatory markers during the first year of disease evolution differed according to the disease course, with patients in the persistent group having significant higher CRP levels at 12 months.

Moreover, use of sDMARD was identified as a predictor of persistent disease evolution in our systemic JIA cohort. In line with that, Beukelman et al. reported, in 2012, a higher use of methotrexate and ciclosporin in systemic JIA patients with polyarthritis and children with radiologic damage, respectively [14]. Therefore, an important use of sDMARDs in patients with persistent disease course could retrospectively be interpreted as an indirect sign of a greater articular involvement which is expected in this group. The long treatment duration in the persistent group is interlinked with the definition of the group but should also be seen as an evidence of how difficult treatment of these chronically affected children is. The long treatment duration in the polyphasic group is due to mostly several relapses of the disease resulting in a therapeutic intervention. We also provide evidence that therapeutic strategies remain heterogeneous and are based on exacerbation if the initial essay is not effective. Furthermore, we show that glucocorticoids remain an important baseline therapy; however, biologic agents are faster and more frequently used in the therapeutic procedure due to important scientific evidence of their efficacy and safety in systemic JIA [17–20]. Baris et al. showed that the duration of steroid treatment correlated with the duration from disease onset to first bDMARDs initiation, mostly related with anakinra and canakinumab [15]. Anti-IL-1 agents (anakinra, *n* = 32; canakinumab, *n* = 25) were the most prevalent therapeutic group in the bDMARD category in our study. However, tocilizumab (anti-IL-6R monoclonal antibody, *n* = 33) was highly used as well and was significantly more often prescribed in the persistent group. Without drawing any conclusions about the efficacy of Tocilizumab, one could hypothesize, that one single target therapy may not be enough to achieve inactive disease, as an established disease may result from complex immunological pathways and a multitude of cytokines (e.g. IL-1β, IL-6, IL-18, IL-17) implicated in the inflammatory process of systemic JIA. Therefore, it may be the high complexity of the aberrant innate immune system in this pathology rendering therapy and prediction of disease evolution difficult [21–23].

There are several limitations in this study. One main limitation is the non-standardized retrospective data collection due to limited time and resources within the framework of this study. Therefore, patients included in this study, despite the complete information on disease activity, had nonetheless missing information on medication use, such as non-reported glucocorticoid or *Non-steroidal anti-inflammatory drugs* (*NSAIDs*) therapy, missing information on laboratory values as well as missing PGA and VAS evaluations by patients. Another main limitation is that excluded patients may differ from the included population leading to a potential selection bias, even if the basic socio-demographic and clinical data from baseline were comparable in both included and excluded patients (Table 1). More cases with available information would have increased the statistical power of the multivariable models. However, our univariate findings, with the significantly higher frequency of arthritis and the significantly higher rate of elevated inflammatory markers during the first year post-diagnosis in the chronic disease evolution group, are tendencies that mirror identified predictors by a multiple regression model in previous studies [8, 9, 11, 21].

## Conclusions

In our model parameters like young age at disease onset (< 6 years) and active arthritis at 12 months, were found to potentially have an influence on the risk of a persistent disease evolution during the first 24 months after diagnosis, even if the association was no longer statistically significant after adjusting for confounding factors. The need of sDMARD use may also predict a persistent disease course, reflecting a more severe phenotype. Further research should concentrate on standardized definitions of inactive disease and remission, which would enable combining harmonized data in meta-analyses. Moreover, future JIA research needs to be conducted on a larger sample with stringently prospectively collected data in order to generate enough statistical power to confirm or reject the presented tendencies within this study.

### Supplementary Information

**Additional file 1: Supplementary Table 1.** Clinical and laboratory features of patients with persistent vs non persistent disease evolution.

## Data Availability

The datasets used and/or analysed during the current study are not publicly available due to individual privacy but are available from the corresponding author on reasonable request.

## Abbreviations

bDMARD: Biologic disease modifying anti-rheumatic drugs
CRP: C-reactive protein
ESR: Erythrocyte sedimentation rate
JIA: Juvenile idiopathic arthritis
NSAIDs: Non-steroidal anti-inflammatory drugs
OR: Odds Ratios
PGA: Physician’s global assessment
sDAMRD: Synthetic disease modifying anti-rheumatic drugs
VAS: Visual analogue scale
