# Supplementary material for: Disease evolution in systemic juvenile idiopathic arthritis: an international, observational cohort study through JIRcohort
Source: Pediatr Rheumatol Online J. 2023 Sep 7;21:96. doi: 10.1186/s12969-023-00886-9 (PMC10485973; doi:10.1186/s12969-023-00886-9)
Supplement: Supplementary file 1 — Additional file 1: Supplementary Table 1. Clinical and laboratory features of patients with persistent vs non persistent disease evolution. [file 12969_2023_886_MOESM1_ESM.docx]

**Supplementary Materials:**

**Supplementary Table 1*:*** Clinical and laboratory features of patients with persistent vs non persistent disease evolution*.*

|  | **6 months post-diagnosis** | | | **12 months post-diagnosis** | | |
| --- | --- | --- | --- | --- | --- | --- |
| **Clinical/laboratory characteristics** | **Non persistent disease evolution**  **(%)** | **Persistent disease evolution***  **(%)** | **p-value** | **Non persistent disease evolution**  **(%)** | **Persistent disease evolution***  **(%)** | **p-value** |
| **Arthritis**  **Oligoarthritis**  **Polyarthritis** | 10  7  3 | 17  8  8 | 0.301  0.917  0.633 | 7^A^  7 ^A^  0 | 45**  31  14 | 0.001  0.114  0.999 |
| **Fever** | 7 | 0 | 0.999 | 0 | 11 | 0.999 |
| **Rash** | 7 | 6 | 0.961 | 0 | 19 | 0.999 |
| **Lymphadenopathy** | 3 | 3 | 0.972 | 0 | 6 | 0.999 |
| **Hepatomegaly** | 0 | 0 | - | 0 | 3 | 1.000 |
| **Splenomegaly** | 0 | 0 | - | 0 | 0 | - |
| **Serositis** | 0 | 3 | 1.000 | 0 | 3 | 1.000 |
| **ESR ≥ 26 mm/h** | 10 | 14 | 0.359 | 14 | 19 | 0.509 |
| **CRP > 10 mg/L** | 21 | 14 | 0.790 | 7 | 47 | < 0.001 |
| **PGA [max score 10], median (25^th^ – 75^th^ percentile**) | 0 (0 – 3.1) | 2 (0 – 6) | 0.288 | 0 (0 – 0) | 5 (2 – 7) | < 0.001 |
| **VAS [max score 10] by patient, median (25^th^ – 75^th^ percentile**) | 0 (0 – 0) | 7 (1 – 8) | 0.999 | 0 (0 – 0) | 4.5 (2.2 – 6) | 0.999 |

Univariate logistic regression comparing the persistent to the non persistent disease evolution at different time points. ^A^ 2 patients from the polyphasic group. *Persistent disease evolution: persistent disease during 24 months. ESR; erythrocyte sedimentation rate, CRP; C-reactive protein, PGA; Physician’s Global Assessment, VAS; visual analogue scale.
